# The nascent polypeptide-associated complex (NAC) controls translation initiation *in cis* by recruiting nucleolin to the encoding mRNA

**DOI:** 10.1093/nar/gkac751

**Published:** 2022-09-15

**Authors:** Alice J L Zheng, Aikaterini Thermou, Chrysoula Daskalogianni, Laurence Malbert-Colas, Konstantinos Karakostis, Ronan Le Sénéchal, Van Trang Dinh, Maria C Tovar Fernandez, Sébastien Apcher, Sa Chen, Marc Blondel, Robin Fahraeus

**Affiliations:** Inserm UMRS 1131, Institut de Génétique Moléculaire, Université de Paris, Hôpital St. Louis, F-75010 Paris, France; Inserm UMRS 1131, Institut de Génétique Moléculaire, Université de Paris, Hôpital St. Louis, F-75010 Paris, France; ICCVS, University of Gdańsk, Science, ul. Wita Stwosza 63, 80-308 Gdańsk, Poland; Inserm UMRS 1131, Institut de Génétique Moléculaire, Université de Paris, Hôpital St. Louis, F-75010 Paris, France; ICCVS, University of Gdańsk, Science, ul. Wita Stwosza 63, 80-308 Gdańsk, Poland; Inserm UMRS 1131, Institut de Génétique Moléculaire, Université de Paris, Hôpital St. Louis, F-75010 Paris, France; Inserm UMRS 1131, Institut de Génétique Moléculaire, Université de Paris, Hôpital St. Louis, F-75010 Paris, France; Inserm UMR 1078, Université de Bretagne Occidentale (UBO), Etablissement Français du Sang (EFS) Bretagne, CHRU Brest, 29200, Brest, France; Inserm UMR 1078, Université de Bretagne Occidentale (UBO), Etablissement Français du Sang (EFS) Bretagne, CHRU Brest, 29200, Brest, France; Inserm UMRS 1131, Institut de Génétique Moléculaire, Université de Paris, Hôpital St. Louis, F-75010 Paris, France; ICCVS, University of Gdańsk, Science, ul. Wita Stwosza 63, 80-308 Gdańsk, Poland; Institut Gustave Roussy, Université Paris Sud, Unité 1015 département d’immunologie, 114, rue Edouard Vaillant, 94805 Villejuif, France; Department of Medical Biosciences, Building 6M, Umeå University, 901 85 Umeå, Sweden; Inserm UMR 1078, Université de Bretagne Occidentale (UBO), Etablissement Français du Sang (EFS) Bretagne, CHRU Brest, 29200, Brest, France; Inserm UMRS 1131, Institut de Génétique Moléculaire, Université de Paris, Hôpital St. Louis, F-75010 Paris, France; Department of Medical Biosciences, Building 6M, Umeå University, 901 85 Umeå, Sweden; RECAMO, Masaryk Memorial Cancer Institute, Zluty kopec 7, 65653 Brno, Czech Republic

## Abstract

Protein aggregates and abnormal proteins are toxic and associated with neurodegenerative diseases. There are several mechanisms to help cells get rid of aggregates but little is known on how cells prevent aggregate-prone proteins from being synthesised. The EBNA1 of the Epstein-Barr virus (EBV) evades the immune system by suppressing its own mRNA translation initiation in order to minimize the production of antigenic peptides for the major histocompatibility (MHC) class I pathway. Here we show that the emerging peptide of the disordered glycine–alanine repeat (GAr) within EBNA1 dislodges the nascent polypeptide-associated complex (NAC) from the ribosome. This results in the recruitment of nucleolin to the GAr-encoding mRNA and suppression of mRNA translation initiation *in cis*. Suppressing NAC alpha (NACA) expression prevents nucleolin from binding to the GAr mRNA and overcomes GAr-mediated translation inhibition. Taken together, these observations suggest that EBNA1 exploits a nascent protein quality control pathway to regulate its own rate of synthesis that is based on sensing the nascent GAr peptide by NAC followed by the recruitment of nucleolin to the GAr-encoding RNA sequence.

## INTRODUCTION

The formation and persistence of protein aggregates is associated with numerous neurodegenerative diseases, including Alzheimer's disease with the development of β-amyloid plaques, or in Huntington's disease where the synthesis of polyQ stretches in the huntingtin protein causes the formation of assemblies through weak side-by-side interactions (1). Apart from β-amyloids, aggregates are predominantly composed of misfolded or unfolded proteins (2). Upon acute stress, cells accumulate unfolded proteins that are cleared by different cellular pathways, including autophagy, secretion in exophers, or interactions with heat shock proteins (HSPs). HSPs prevent the formation of aggregates by supporting the proper folding or engaging with misfolded or aggregate-prone proteins for refolding or to target them for degradation (2–4). Ribosome stalling leads to co-translational ubiquitination and degradation of the nascent polypeptide via the ribosome-associated protein quality control (RQC) pathway (5–8) but how the cell senses and prevents the synthesis of aggregate-prone proteins is still relatively unknown.

The nascent polypeptide-associated complex (NAC) is present at the ribosome exit tunnel and is composed of NAC alpha (NACA) and BTF3 (also called NAC beta or NACB) (9,10). NAC is suggested to be the first chaperone the nascent polypeptide encounters and crystallography studies show that the BTF3 N-tail is present within the exit tunnel and escorts the nascent polypeptide (11). It interacts with short nascent polypeptide sequences and protects them from proteolysis and from interacting with other nascent polypeptide-associated factors, such as the SRP (signal recognition particle) and the methionine aminopeptidase (12,13). The flexible conformation of NAC allows it to interact with a wide range of substrates and NAC chaperone activity has been shown to prevent aggregate accumulation outside of its ribosome-bound state (14–16).

mRNA translation initiation is a tightly regulated dynamic process governed by multiple signalling pathways and factors to allow a selection of mRNAs to be translated depending on cell type and conditions (17,18). RNA-binding proteins (RBPs) are adaptable regulators that interact with RNA sequences, or with RNA structures, and play a key role in guiding translation (19,20). It has also been suggested that translation drives the formation of RNA structures (21). The binding of nucleolin (NCL) to G-quadruplex (G4) structures in the glycine-alanine repeat (GAr) domain of the Epstein-Barr virus (EBV)-encoded *EBNA1* mRNA forms an essential part in the viral immune evasion strategy by suppressing translation initiation *in cis* in order to minimize the production of antigenic peptides for the major histocompatibility (MHC) class I pathway (22–25). The GAr-encoded peptide forms an aggregate-prone disordered domain consisting of single alanines separated by one, two, or three glycines and varies in length depending on viral strain but can reach over 200 amino acids (Supplementary Table S1). The GAr-encoding mRNA suppresses mRNA translation initiation of every open reading frame to which it is fused and introducing serine residues in every eight amino acids alleviates GAr-mediated inhibition of translation (24,26).

Here, we have used the GAr to show how a nascent disordered peptide acts together with G4 structures within the encoding RNA to control mRNA translation initiation *in cis*. Our results suggest that in order to minimise the synthesis of antigenic peptides, EBNA1 exploits a nascent protein quality control pathway that serves to prevent the synthesis of aggregate-prone proteins and is based on the dislodgment of NAC by the nascent peptide.

## MATERIALS AND METHODS

### Cell culture

Human carcinoma-derived cell line H1299, EBV carrying Burkitt's lymphoma cell line Raji, the *in vitro* transformed B95.8 and nasopharyngeal carcinoma cell line NPC 666-1 were all cultivated under standard conditions in RPMI 1640 medium containing 10% fetal calf serum, 2 mM l-glutamine, and 100 IU/ml penicillin and streptomycin (Gibco-BRL). NPC 666-1 was a kind gift from Pr. Kwok-Wai Lo from the Chinese University of Hong Kong.

### RNA G4 structures and solubility prediction of the polypeptides

RNA G4 structures predictions were realised using the webserver QGRS mapper (27). Solubilities of the polypeptides for *E.coli* expression were predicted using the webserver SoluProt v1.0 (28).

### Western blotting

Cells were harvested 40h post-transfection and lysed in the presence of a complete protease inhibitor cocktail (Roche Diagnostics). Total cell extracts were fractionated by SDS-PAGE, transferred to BioTrace^®^NT nitrocellulose blotting membranes (Pall Corp.). After incubation with the appropriate primary antibodies and peroxidase-conjugated secondary antibodies (Dako), proteins were visualized by using Pierce ECL, WestDura, or West Femto (ThermoFisher) and quantified with the MyECL Imager system (Thermo Scientific) and ImageJ. The following primary antibodies were used: mouse monoclonal anti-EBNA1 antibody (OT1X, Cyto-Barr), mouse monoclonal anti-p53 antibody (DO-1), mouse polyclonal anti-HA antobody (provided by Dr B. Vojtěšek, Masaryk Memorial Cancer Institute, Brno, Czech Republic), rabbit polyclonal anti-NACA antibody (Abcam), anti-Ovalbumin whole serum (Sigma), rabbit polyclonal anti-NCL antibody (Abcam), rabbit polyclonal anti-LC3B antibody (Sigma) and mouse monoclonal anti-actin antibody (Sigma). Relative quantifications of the HRP signals normalised with the corresponding actin bands are mentioned above the band.

**RNA extraction, RT-qPCR, and RNA *in vitro* co-IP assay**

Cells were washed in cold PBS and total RNA extraction was performed using RNAeasy Mini Kit (Qiagen), following the manufacturer's instructions. cDNA synthesis was carried out using the Moloney murine leukaemia virus M-MLV reverse transcriptase and Oligo(dT)_12-18_ primer (Life technologies). qPCR was performed using the StepOne real-time PCR system (Applied Biosystems) with Perfecta SYBR Green FastMix (Quanta Biosciences).

*In vitro* RNA co-IP was carried out as described elsewhere (29). Briefly, 1μg of total RNA extracted from cells was co-incubated under agitation with 100 ng of recombinant NCL (provided by Dr. M.-P. Teulade-Fichou, Institut Curie, Paris, France) in binding buffer (50 mM Tris pH7.5, 150 mM NaCl, 0.02 mg/ml yeast tRNA, 0.2 mg/ml BSA) for 15 min at 37°C. After incubation, NCL-RNA complexes were pulled down at 4°C using G-coated sepharose beads (Sigma) with anti-NCL rabbit polyclonal antibody (Abcam) according to standard conditions and purified using the TRIzol (Life Technologies). Precipitated RNAs were then analysed by RT-qPCR.

### MHC class I restricted antigen presentation

T-cell assays using the B3Z SIINFEKL:Kb-specific T cell hybridoma were carried out as described previously (30). Briefly, B3Z SIINFEKL:Kb-specific T cell hybridoma were co-cultured with H1299 cells transfected with both Kb and the reporter construct, or with the empty vector (EV), for 20 h. B3Z CD8+ T cell hybridoma expresses LacZ in response to activation of T cell receptors specific for the ovalbumin's immuno-dominant SIINFEKL peptide in the context of H-2Kb MHC class I molecules.

The cells were harvested and washed 2 times with 1X cold PBS before lysis in 0.2% TritonX-100, 0.5M K_2_HPO_4_, 0.5M KH_2_PO_4_ for 5 min on ice. Supernatants from each condition were transferred into 96-well optiplate counting plates (Packard Bioscience, Randburg, SA) and incubated for 1 h at room temperature, protected from light, and tested for β-galactosidase activity using the Luminescence assay (BD Biosciences Clontech) on a FLUOstar OPTIMA (BMG LABTECH Gmbh, Offenburg, Germany). The results are expressed in luminescence units.

### RNA FiSH

H1299 cell were seeded in 24-well plates (2 x 100 cells/ well) and trasient transfections were carried out 24 h later using the Genejuice reagent (Merck Biosciences) according to the manufacturer's protocol. 24 h after transfection the cells were briefly washed with ice-cold PBS, fixed with 4% PFA during 20 minutes at room temperature, and washed again with PBS. Cell were then incubated in 70% ethanol for 4-24 h at 4°C. For rehydration, the cells were incubated in 50% and 30% ethanol and further washed with PBS. Subsequently, cells were permabilised with PBS 0.4% Triton 0.05% CHAPS for 5 minutes at room temperature. Coverslips were incubated overnight in a wet chamber at 37°C in FiSH hybridisation buffer supplemented with 10% dextran sulphate and 100nM of FiSH Stellaris probes targeting Ovalbumin or EBNA1 mRNAs (Biosearch Technologies). Coverslips were washed twice 20 min in FiSH hybridisation buffer and 5 min in FiSH Wash buffer and subsequently stained with DAPI. Images were obtained using Zen software (Zeiss) and the Costes colocalisation factor between nuclei (DAPI channel) and targeted mRNAs (Cy3 channel), called Correlation_Costes_Cy3_DAPI, were obtained using the software CellProfiler (31), and later used for statistical analysis.

### Proximity ligation assay (PLA) protein–protein and immunostaining

Cells were cultured, fixed, and permeabilised as described above. Primary antibodies incubation and PLA were carried out using the Naveniflex MR kit (Navinci), following the manufacturer's protocol. The aggregates staining were realised using the Proteostat Aggresome detection kit (Enzo) following the manufacturer's instructions. PLA antibodies: mouse polyclonal anti-HA antibody, rabbit polyclonal anti-NACA antibody (Abcam), rabbit polyclonal anti-HA antibody (Sigma), mouse polyclonal anti-NACA antibody (Abnova) and mouse monoclonal anti-EBNA1 antibody (OT1X, Cyto-Barr). Images were obtained using Zen software and analysed using CellProfiler. Data were processed by taking into account the difference in targeted protein expression, meaning the number of PLA dots were normalised with the corresponding cells integrated immunofluorescence signal measured, and then the mean relative difference between the different conditions was calculated.

### Protein co-immunoprecipitation (co-IP)

After centrifugation, cell pellets from Raji or B95.8 cultures were lysed in buffer containing 20 mM Tris (pH 7.5), 150 mM NaCl, 1% NP-40 in the presence of complete protease inhibitor cocktail (Roche). Lysates were immunoprecipitated with anti-EBNA1 goat antibody or goat IgG and protein G-sepharose. The beads were washed with PBS and lysis buffer x4 and boiled in SDS loading buffer. Immunoprecipitates were analysed by SDS/PAGE using 4–12% pre-cast gels (Invitrogen).

### Polysome fractionation and PLEA

5–50% (w/vol) linear sucrose gradients were freshly cast on SW41 ultracentrifuge tubes (Beckmann) using the Gradient master (BioComp instruments) following the manufacturer's instructions. Polysome fractions were collected and concentrated to 100 μl using the Millipore concentrating falcon tubes. Protein concentration was measured by Bradford and an equal amount of protein from each sample was used for the PLEA experiment to study the interaction of three molecules. 96-well ELISA plates were incubated with the capture antibody at a dilution of 1:200, o/n, at 4°C. Samples were incubated with the PLA secondary antibodies and the PLA kit (Sigma) was used according to manufacturers’ instructions. The fluorescence at 640 nm was measured by the FLUOstar plate reader (excitation at 644 nm and emission at 669 nm). The values were used for the preparation of a graph and for statistical analysis to calculate the mean and the standard deviation using the GraphPad Prism 9 software. Each sample was tested in triplicates.

### RNA pulldown assays

For the preparation of whole-cell extracts, confluent H1299 cells were collected after trypsin treatment and washed twice with 1X PBS (Gibco). Cells were suspended in 500 μl of lysis buffer (20 mM Tris–HCl pH 7.5; 200 mM NaCl and 0.1% Igepal) containing 1× protease inhibitor cocktail (Roche). Cell lysis was performed by five series of vortex followed by 10 min incubation on ice, and 3 series of 3s sonication at 20% amplitude. After lysis cells were centrifuged at 4°C for 15 min at 16 000g, and the supernatant was quantified by Bradford. The whole-cell extracts or recombinant GST-NCL (Abnova) and His-tagged NACA (homemade, Umea University) were used for pulldown assays with the following G-quadruplex forming oligonucleotides: GQ-18 5′-GGGGCAGGAGCAGGAGGA-3′Biotin TEG. The negative control for EBNA1 G4 was the GM-18 5′ GAGGCAGUAGCAGUAGAA-3′Biotin TEG oligonucleotide which, according to the QGRS mapper software (Ramapo College New Jersey https://bioinformatics.ramapo.edu/QGRS/credits.php), is unable to form G4 structures. To avoid unspecific binding, high-affinity streptavidin sepharose beads (GE Healthcare) were incubated in 1 ml blocking buffer containing 10 mM Tris–HCl pH 7.5; 100 mM KCl; 0.1 mM EDTA; 1 mM DTT; 0.01% Triton X-100; 0.1% BSA; 0.02% *S. cerevisiae* tRNAs (Sigma), for 1 h at 4 °C on a rotating wheel. An amount of 10 pg of each folded biotinylated RNA oligos was incubated with 50 μl of the solution containing the streptavidin sepharose beads for 90 min at 4°C on a rotating wheel. Five hundred micrograms of cell extract were incubated with the RNA oligonucleotides bound to the streptavidin beads for 90 min at room temperature. Beads were washed with increasing KCl concentration (200–800 mM). Protein still bound to beads after the washes were eluted using 2× SDS loading buffer and analysed by WB against NACA or NCL, as previously described. In the input lane of the WBs was loaded a fraction of the extract that was incubated with the beads for each condition.

### Statistical analysis

Data were analysed by unpaired Mann–Whitney's test or Student's *t*-test on GraphPad Prism 9. On graphs, represented data are the mean and the standard deviation or SEM of a minimum of three independent experiments. Statistical significance of the difference is stated as following: *P* > 0.05 (ns), *P* < 0.05 (*), *P* < 0.01 (**) and *P* < 0.001 (***).

## RESULTS

### NAC controls the synthesis of GAr-containing polypeptides

**Figure 1.**
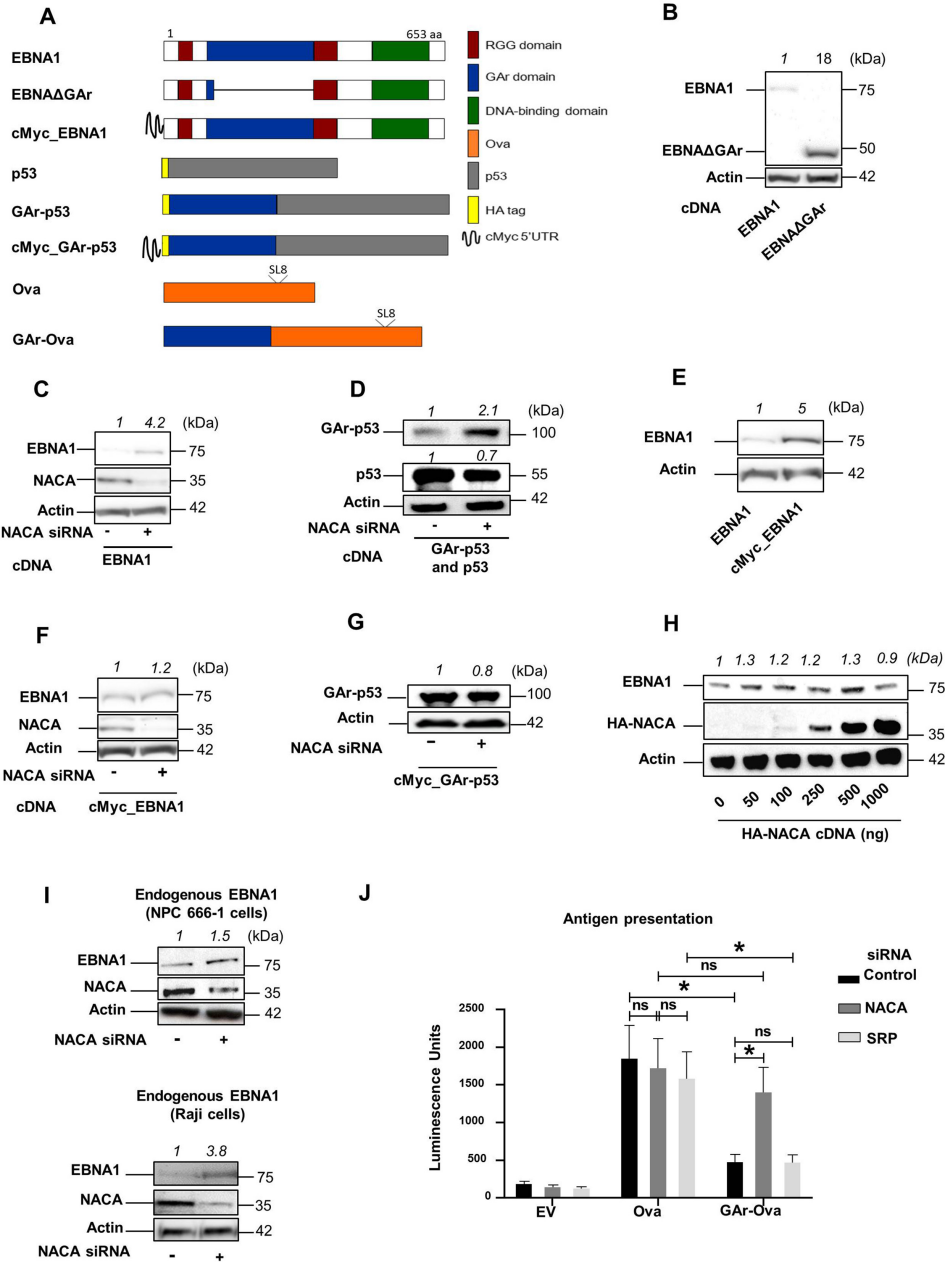
(**A**) Cartoon illustrating the constructs used. Position of Ova-derived SIINFEKL antigenic MHC class I peptide (SL8) is indicated. (**B**) Western Blot (WB) from H1299 cells transfected with EBNA1 cDNA or an EBNA1 that lacks the gly-ala repeat (EBNAΔGAr). (**C**) WB from H1299 cells transfected with EBNA1 and treated with siRNAs control (-) or with siRNA against NACA (NAC subunit alpha) (+). (**D**) WB from H1299 cells transfected with GAr-p53 or p53 constructs and treated with indicated siRNAs. (**E**) WB from H1299 cells transfected with EBNA1 or an EBNA1 construct carrying *cMyc* in its 5′ UTR (cMyc_EBNA1). (**F**) WB from H1299 cells expressing cMyc_EBNA1 and treated with indicated siRNAs. (**G**) WB of H1299 expressing the cMyc_GAr-p53 construct and treated with indicated siRNAs. (**H**) WB of H1299 cells transfected with an EBNA1 construct and increasing amounts of HA-NACA-encoding plasmid. (**I**) WB of endogenous EBNA1 in EBV-carrying NPC 666-1 cells (upper panel) or Raji cells (lower panel) treated with indicated siRNAs. (**J**) Antigen presentation assay performed using B3Z SIINFEKL:Kb-specific T cell hybridoma co-cultured with H1299 cells transfected with Ova, GAr-Ova or empty vector (EV) and treated with siRNAs against NACA or SRP. Quantification of WBs is indicated on the top of each immunoblot. Proteins detected are indicated on the left of the immunoblots. Data represent three independent experiments.

The gly-ala repeat (GAr) domain is located in the N-terminal part of the EBNA1 and deleting this domain (EBNAΔGAr) increases mRNA translation and protein expression (Figure 1A and B) (23–26,32). Previous studies have shown that the binding of nucleolin (NCL) to the *EBNA1* mRNA is important for translation suppression *in cis* (22). However, antibodies against the GAr domain of EBNA1 overcome translation suppression *in vitro* (23), showing that the peptide also plays a role in controlling translation initiation. We wanted to know how the mRNA and the encoded peptide both play a role in controlling EBNA1 synthesis. In order for both the RNA and the encoded peptide to control translation initiation *in cis*, we reasoned that the mechanism of action should take place before the protein leaves the translating ribosome. NAC is described as a ribosome-associated chaperone targeting aggregate-prone proteins and we tested the impact of NAC on GAr peptide-mediated protein synthesis control. siRNA-mediated silencing of NAC subunit alpha (NACA) in H1299 cells leads to a decrease in *NACA* mRNA and protein levels and this resulted in an increase of protein aggregates and an increase in LC3 protein and polyQ-fused protein levels, showing that the reduced expression resulted in a functional response (Figure 1C and Supplementary Figures S1A, S1B and S1C) (16,33).This was accompanied by an approximately 4-fold increase in EBNA1 expression without affecting *EBNA1* mRNA levels (Figure 1C and Supplementary Figure S1D). A similar increase in expression following NACA knockdown was also observed when GAr was fused to the N-terminus of p53 (GAr-p53) while only a limited, or no, effect was observed on p53 alone (Figure 1D). Fusing the GAr to ovalbumin (Ova) (GAr-Ova) also resulted in a NACA-dependent expression control (Supplementary Figure S1E). These results show that silencing NACA interferes with GAr-mediated translation inhibition. In addition, the GAr is known to be more efficient in suppressing translation when placed in the 5′ of the coding sequence, as compared to the 3′ (23,26), and NACA silencing had less impact when GAr was fused to the 3′ of the Ova (Ova-GAr), supporting the notion that NACA acts on GAr-mediated translation control (Supplementary Figure S1E). To further test that the effect of NACA on EBNA1 expression is at the level of mRNA translation, we took advantage of the fact that fusing the 5′ untranslated region (UTR) of *c-Myc* to the 5′ UTR of EBNA1 (cMyc_EBNA1) overcomes GAr-mediated translation inhibition (Figure 1E) (26,32,34). When we knocked out NACA we observed no effect on the expression of EBNA1 carrying the *cMyc* sequence in the 5′ UTR (Figure 1F). Similarly, when we fused the *cMyc* sequence to the 5′ UTR of GAr-p53 or GAr-Ova constructs, we did not observe any increase in expression following NACA knockdown, validating that the effect of NACA is indeed on the synthesis of EBNA1 and not on protein stability (Figure 1G and Supplementary Figure S1F). Overexpressing an HA-tagged NACA did not affect EBNA1 expression, indicating that the endogenous NACA levels are sufficient to control EBNA1 synthesis (Figure 1H). To ensure that endogenous EBNA1 expression is controlled by NAC, we also treated the EBV-carrying NPC 666-1 and Raji cells with siRNA against NACA and we observed a similar increase in EBNA1 expression (Figure 1I and Supplementary Figure S1G). The physiological role of the GAr is to minimise the production of EBNA1-derived peptide substrates for the MHC class I pathway and we wanted to know if NACA affects the production of antigenic peptide substrates. The Ova includes an antigenic peptide (SL8; see Figure 1A) that is presented on the murine Kb class I molecules. When we expressed Ova, or GAr-Ova, and exposed the cells to B3Z CD8 + T cells that are specific for SL8, we observed an increase in antigen presentation following NACA siRNA treatment in cells expressing GAr-Ova but not in cells expressing Ova. Silencing SRP (Signal Recognition Particle), another nascent polypeptide-associated factor, had no impact on antigen presentation (Figure 1J). Taken together, these results show that NAC plays a key role in GAr-mediated suppression of mRNA translation initiation and in the viral strategy to evade the immune system.

### NAC interacts with the GAr peptide domain

**Figure 2.**
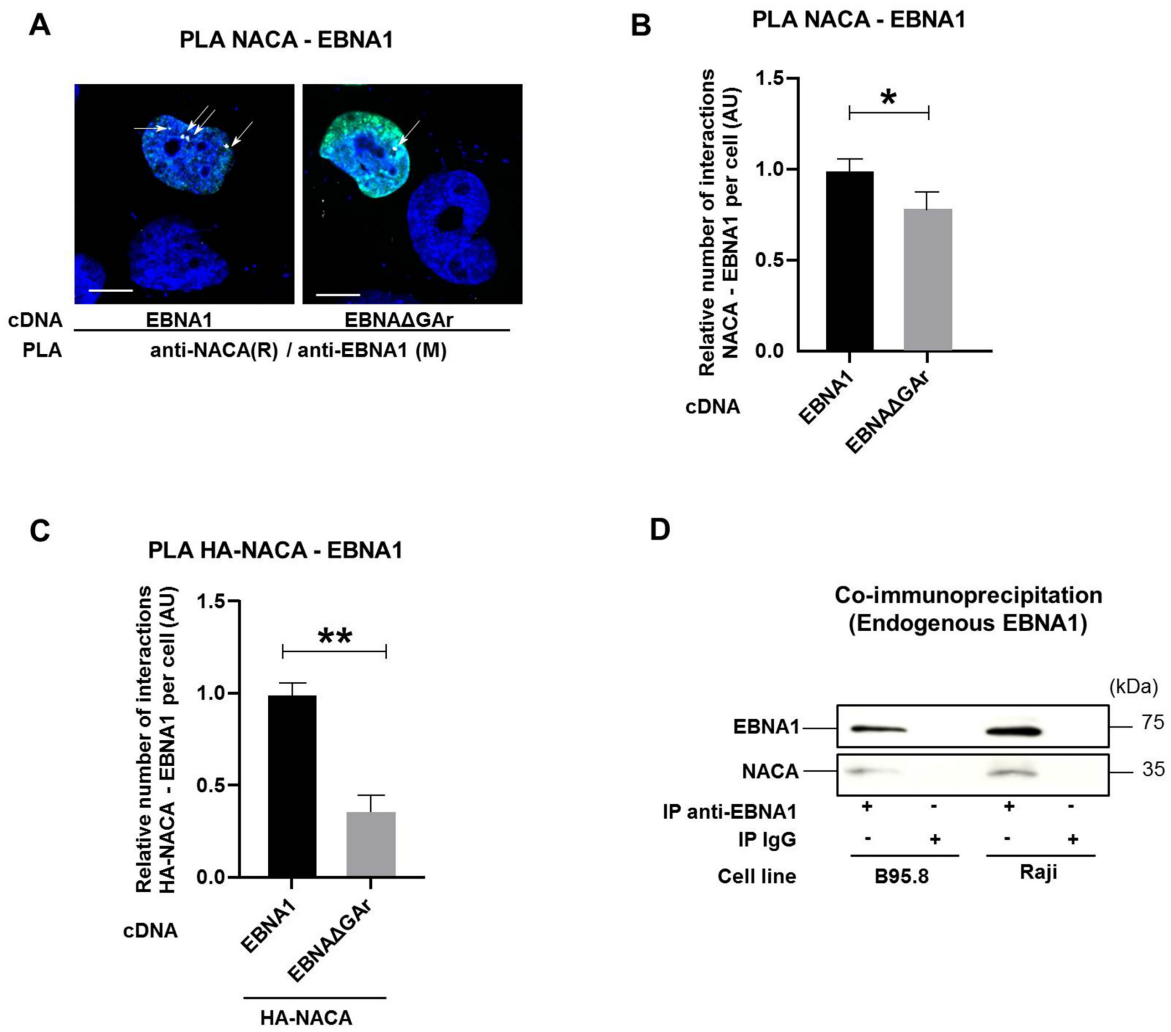
(**A**) Proximity ligation assay (PLA) (white arrows) shows interactions in H1299 cells between NACA and EBNA1 or NACA and EBNAΔGAr. Co-immunofluorescence of respective proteins shown in green. DAPI nuclear staining in blue. Scale bar represents 10 μm. (**B**) Relative number of interactions of indicated reporter proteins with endogenous NACA detected by PLA and normalised with the expression of the corresponding reporter proteins in H1299 cells expressing EBNA1 or EBNAΔGAr. (**C**) Relative number of interactions between an exogenous HA-tagged NACA and EBNA1 or EBNAΔGAr. (**D**) Co-IP using anti-EBNA1 or anti-IgG (negative control) antibodies in EBV-carrying B95.8 and Raji cell lysates. Data represent three independent experiments. A minimum of 50 cells expressing indicated reporter protein was counted for each PLA experiment.

To address how NAC mediates GAr-dependent translation control, we performed Proximity Ligation Assay (PLA) to see if endogenous NACA interacts with EBNA1 or the EBNAΔGAr *in situ*. We observed fewer interactions with the EBNAΔGAr, as compared to EBNA1, despite EBNAΔGAr being expressed at four times higher levels. The interactions were mainly observed in the nuclear compartment (Figure 2A). As a control we used the HA-polyQ-p53 as poly-glutamine repeat (PolyQ) causes aggregates and is known to interact with NAC (Supplementary Figure S2A). In line with previous studies, immunofluorescence assays confirmed a cytoplasmic and nuclear localisation of NACA (Supplementary Figure S2B) (35). After taking into account the difference in expression levels between EBNA1 and EBNAΔGAr (see Figure 1B), we estimated that deleting the GAr significantly decreased the number of interactions between endogenous NACA and EBNA1 peptide (Figure 2B). Overexpressing an HA-NACA construct together with EBNA1, or EBNAΔGAr, showed an approximately 3-fold increase in the number of interactions between NACA and EBNA1 peptide, as compared to NACA and EBNAΔGAr (Figure 2C). Co-immunoprecipitation assays from two EBV-carrying B cell lines (B95.8 and Raji) showed that endogenous EBNA1 protein interacts with NACA (Figure 2D). We also wanted to see if NACA interacts with the newly synthesised EBNA1 peptide and we therefore performed a metabolic labelling assay using S35 methionine in order to visualise newly synthesised polypeptides that co-immunoprecipitate with NACA. When comparing control cells (EV) with cells transfected with EBNA1, we observed a band corresponding to the size of EBNA1 in cell lysates immunoprecipitated with NACA antibodies. The same size band was detected in Western Blot (Supplementary Figure S2C). These results indicate that NAC interacts with the newly synthesized GAr peptide.

### The nascent GAr peptide dislodges NAC from the ribosome

**Figure 3.**
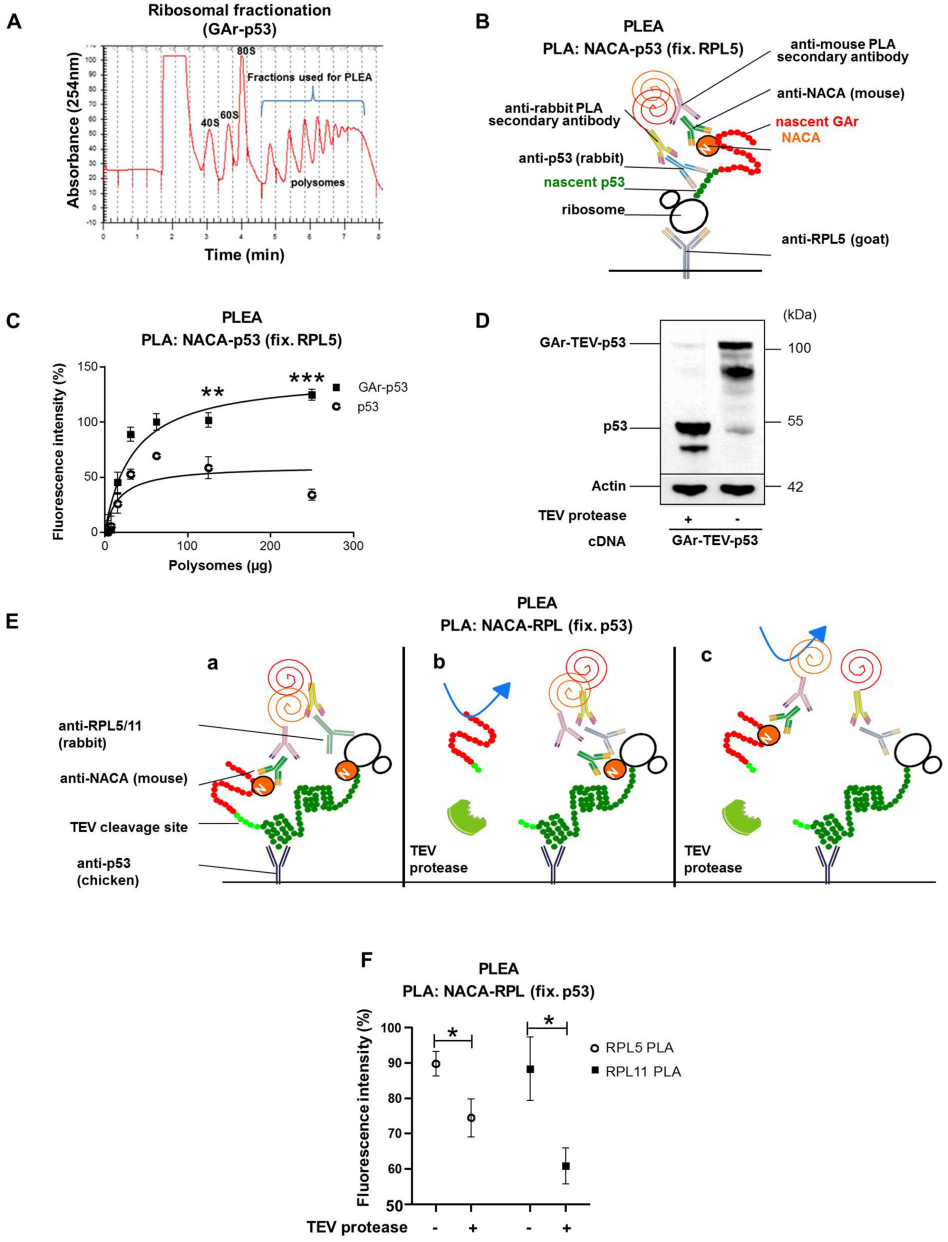
(**A**) Ribosomal fractionation of H1299 cells. Polysomal fractions collected and used for the PLEA experiments are indicated in blue. (**B**) Cartoon illustrating the PLEA experiment. Polysomes were fixed with a goat anti-RPL5 antibody and the PLA was performed on the captured polysomes and their associated complex using anti-NACA (M = mouse) and anti-p53 (R = rabbit) antibodies. (**C**) The graph shows PLEA results where more interactions between p53 and NACA were detected on GAr-p53, as compared to p53, expressing polysomes. The data show normalised fluorescence intensity for GAr-p53 and p53 polysomes. T-tests were performed between GAr-p53 and p53 values for the same amount of polysomes. (**D**) WB of H1299 cells transfected with a construct in which the TEV proteolytic cleavage site was inserted between GAr and p53 (GAr-TEV-p53). The lysates were treated, or not, with TEV protease. (**E**) Cartoons illustrate the PLEA experiment to assess if GAr dislodges NACA from the ribosome. (**F**) Polysomes from H1299 cell lysates expressing GAr-TEV-p53 and treated with TEV protease, or not, were captured using a chicken anti-p53 antibody. PLEA experiments were performed using 125μg of polysomes and PLA was carried out using anti-NACA(M = mouse) with anti-RPL5(R = rabbit) antibodies (white circles) or anti-NACA(M) with anti-RPL11(R) antibodies (black squares). The graph shows the relative amount of NACA bound to GAr-TEV-p53 translating ribosomes before and after treatment with TEV protease. The data represent three independent experiments.

We next tested if NAC interacts with the nascent EBNA1 on the ribosome, as this would help explain how the interaction between GAr and NAC controls *EBNA1* translation *in cis*. We expressed p53 and GAr-p53 and carried out polysomal fractionation using sucrose gradients on cycloheximide-treated cell lysates (Figure 3A). The isolated polysomes were tested for the presence of *GAr-p53* mRNA and nascent GAr-p53 derived peptides in order to ensure that they were actively translating the GAr-p53 message (Supplementary Figures S3A and S3B). The polysomes were captured on 96-well plates using goat anti-RPL5 sera. We did an adapted PLA ELISA (PLEA) by adding increasing amounts of polysomes followed by *in vitro* PLA using anti-p53 (rabbit) and anti-NACA (mouse) antibodies (Figure 3B). The fusion of the GAr sequence with p53 resulted in an average 3-fold stronger interaction between the nascent GAr-p53 peptide and NACA, supporting the notion that NAC interacts strongly with the nascent GAr at the riboosme (Figure 3C). To test if the high affinity between GAr and NAC could lead to the dissociation of NAC from the ribosome we designed a construct in which a TEV protease cleavage site was inserted between the GAr and the p53 (GAr-TEV-p53). The GAr peptide will, thus, exit the ribosome and encounter NAC before the TEV and the p53 sequence. To ensure that TEV cleaved the GAr-TEV-p53 fusion protein we added TEV enzyme to cell lysates for 60nbsp;min and we could observe that a major part of GAr was indeed cleaved from p53 (Figure 3D). We then expressed GAr-TEV-p53 and carried out ribosomal fractionations on TEV-treated and non-TEV-treated lysates (Supplementary Figure S3C). p53-carrying polysomes (125 μg) were isolated and fixed to 96-well plates using chicken anti-p53 antibodies and PLEA was performed against ribosomal proteins and NACA (see Figure 3C). If the GAr did not affect NACA’s placement at the ribosome, we would expect that TEV protease treatment would not make any difference on the PLEA signal between NACA and ribosomal proteins (RPL5 or RPL11) (Figure 3E; a and b). However, if the GAr sequence caused NACA to detach from the ribosome, TEV treatment would result in reduced interactions between NACA and RPL5 or RPL11 (Figure 3E; c). The TEV treatment indeed resulted in less PLA signal between NACA - RPL5/RPL11 on GAr-TEV-p53-expressing polysomes, in line with the notion that NACA’s recruitment to the GAr sequence causes NACA to dissociate from the ribosome (Figure 3F). These results show that the nascent GAr peptide interacts strongly with NACA on the ribosome and leads to NACA being dislodged from the ribosome, offering a first insight into how NAC affects mRNA translation of GAr-carrying mRNAs *in cis*.

### NAC is necessary for the interaction between the *GAr*-encoding mRNA and NCL

**Figure 4.**
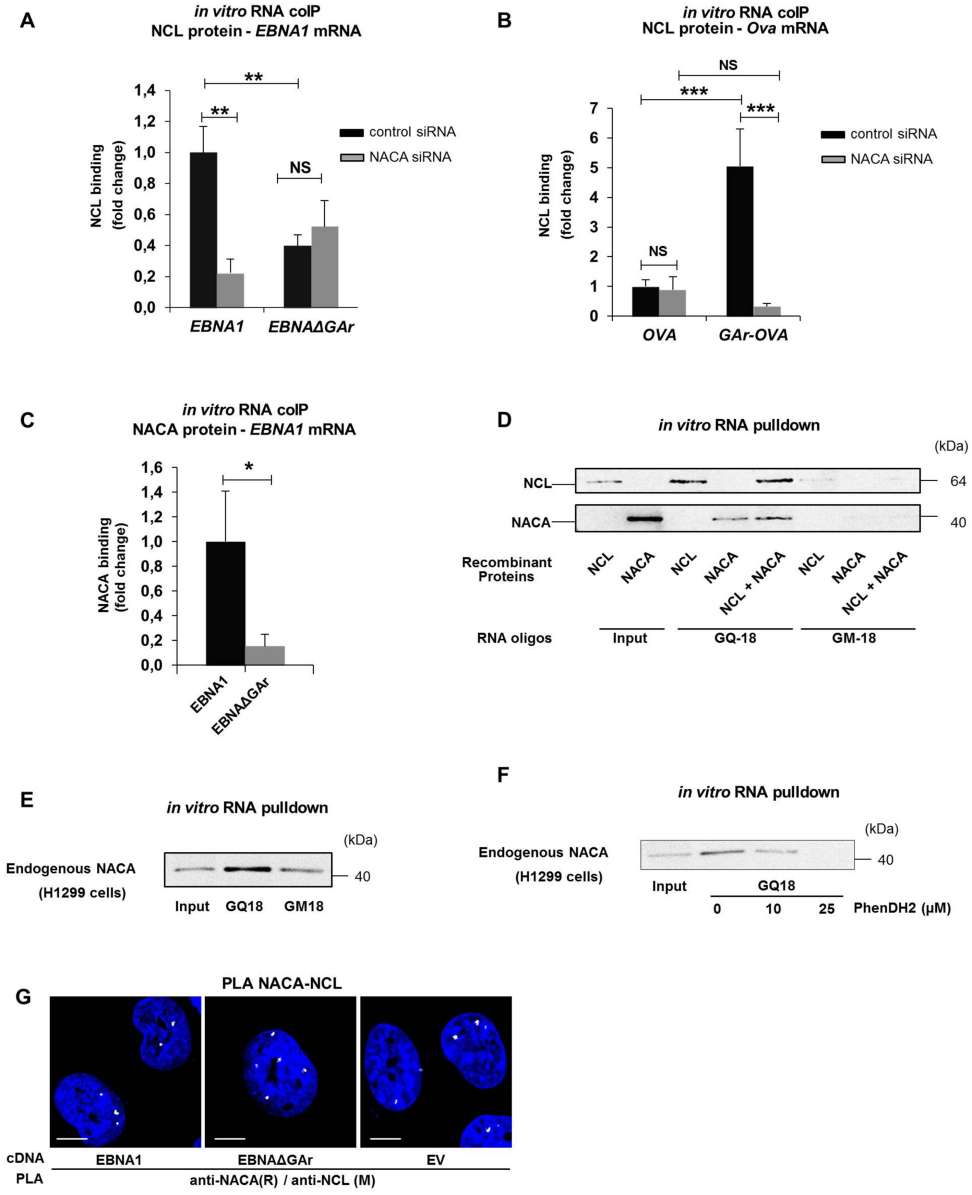
(**A**) *In vitro* RNA coIP experiments performed with recombinant NCL and *EBNA1* or *EBNAΔGAr* mRNA extracted from H1299 cells treated with the indicated siRNAs. The graph shows the fold change in NCL binding. (**B**) *In vitro* RNA coIP performed with recombinant NCL and *Ova* or *GAr-Ova* mRNAs from cells treated with the indicated siRNAs. The graph shows the fold change in NCL binding. (**C**) *In vitro* RNA coIP experiments performed with recombinant NACA and *EBNA1* or *EBNAΔGAr* mRNAs extracted from H1299 transfected cells. The graph shows relative fold change in NACA binding. (**D**) *in vitro* RNA pulldown assay using recombinant NCL and NACA and the indicated RNA oligos. GQ-18 is derived from the G4-forming sequence coding for the GAr. GM-18 is a mutated non-G4-forming version of GQ-18. (**E**) *in vitro* RNA pulldown assay using endogenous NACA from H1299 cells and the indicated RNA oligos. (**F**) *in vitro* RNA pulldown assay using endogenous NACA from H1299 cells, the GQ-18 oligo derived from the G4-forming sequence coding for the GAr and increasing amounts of the PhenDH2 G4 ligand. (**G**) PLA assessing endogenous NACA - NCL interactions in H1299 cells expressing EBNA1 or EBNAΔGAr (EV = empty vector). In blue: nucleus, in white: PLA dots. Scale bar represents 10μm. The data represent a minimum of three independent experiments.

The results show that the nascent GAr peptide plays an important role in GAr-mediated mRNA translation control. However, previous works have demonstrated the importance of the *GAr*-encoding mRNA sequence in suppressing mRNA translation (22,29). The effect of the *GAr* mRNA is attributed to NCL binding G-quadruplex (G4) structures in the *GAr*-encoding mRNA and we wanted to know if there is a link between NAC and the recruitment of NCL to the *GAr* mRNA. To test this, we carried out *in vitro* RNA-protein co-immunoprecipitation (RNA-coIP) assays using recombinant NCL together with RNA isolated from cellular extracts, followed by RT-qPCR for the indicated mRNAs. Silencing NACA resulted in a dramatic decrease in the binding of NCL to the *EBNA1* and *GAr-Ova* mRNAs while no significant change was observed for the already weak interaction between NCL and the *EBNAΔGAr* or *Ova* mRNAs (Figure 4A and B). NAC has been described to bind nucleic acids and we next tested if this capacity can be the link between the *GAr*-encoding RNA sequence and the GAr peptide (10). RNA-coIP using recombinant NACA and mRNAs isolated from cell lysates showed that NACA binds to the *EBNA1* mRNA, but not to the *EBNA1ΔGAr* mRNA (Figure 4C). To test if the NACA – *EBNA1* mRNA interaction is direct, we carried out *in vitro* RNA pulldown assays using recombinant NACA protein together with synthetic oligos forming the RNA G4 structure of the GAr (GQ-18) or a mutated version of GQ-18 that does not form a G4 (GM-18) (22). This showed that NACA binds RNA oligos in a G4-dependent manner (Figure 4D). Interestingly, adding recombinant NCL did not prevent NACA interaction, suggesting that they have different interaction sites (Figure 4D). NACA binding to the G4 forming sequence of the GAr (GQ-18) was further confirmed using endogenous NACA protein pulled down from H1299 cell lysates (Figure 4E). Addition of an increasing amount of the G4 ligand PhenDH2 (32) prevented the interaction of endogenous NACA to the GQ-18, further confirming that NACA binds to the G4 RNA structure of *GAr*-encoding mRNA (Figure 4F). Having observed that both NACA and NCL interact with the *GAr* G4 RNA, we next tested if NACA and NCL proteins interact with each other. *In situ* PLA showed that endogenous NACA and NCL interactions were detected in the nuclear compartment and that this is not limited to EBNA1 expressing cells, as it is also detected in cells transfected with the EBNAΔGAr construct or with the empty vector (EV) (Figure 4G and Supplementary Figure S4A and S4B). These results show that NACA in addition to binding the GAr peptide also interacts with *GAr* mRNA sequence and promotes the NCL–*EBNA1* mRNA interaction.

### Translation is required for the interaction between *GAr*-encoding mRNAs and NCL.

**Figure 5.**
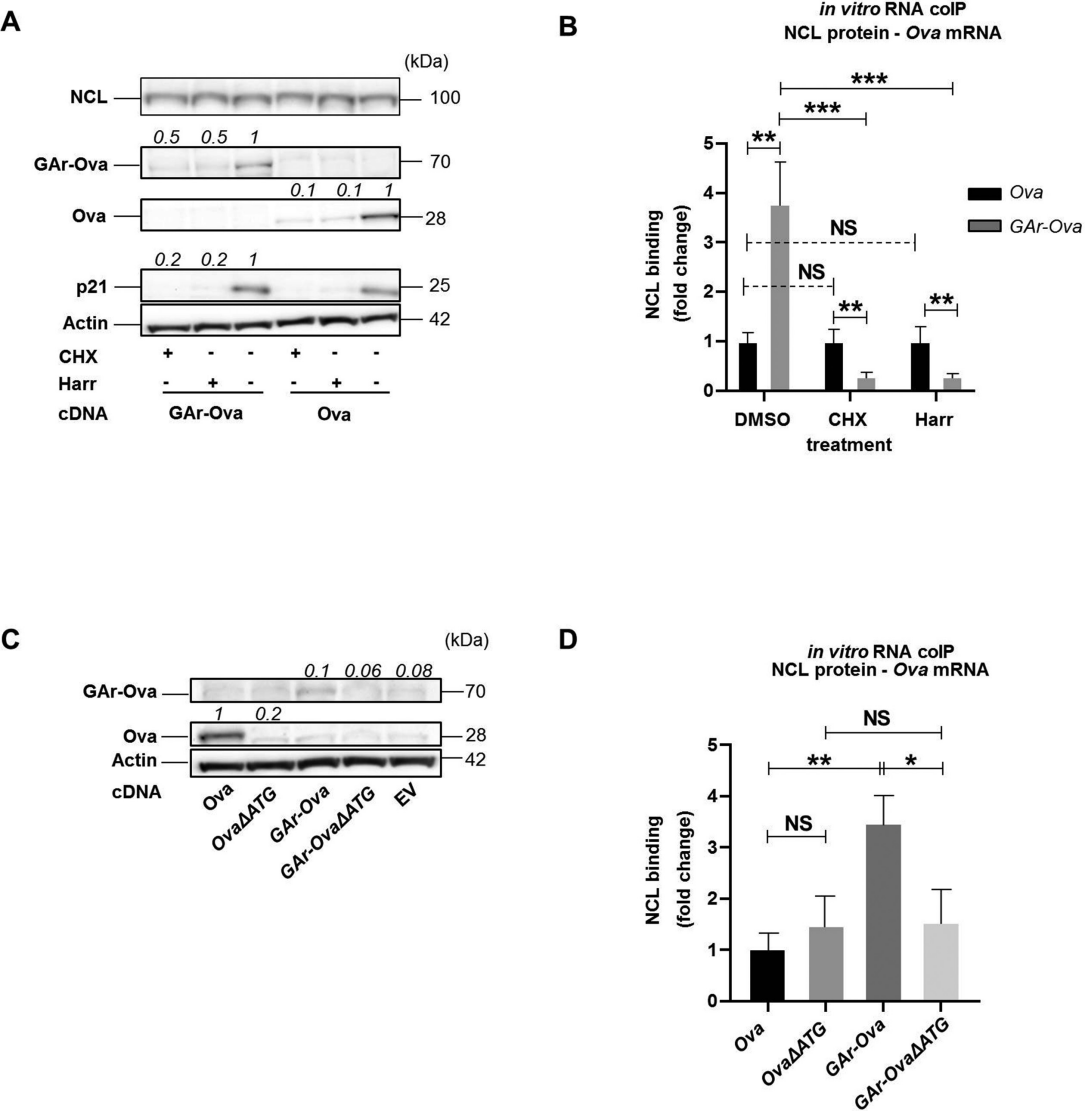
(**A**) WB of lysates from H1299 cells expressing indicated constructs and treated with cycloheximide (CHX), harringtonine (Harr) or DMSO control for 5h. (**B**) *in vitro* RNA coIP experiments performed with recombinant NCL and *Ova* or *GAr-Ova* mRNAs extracted from H1299 transfected cells treated with CHX, Harr or DMSO. The graph shows the relative fold change in NCL binding. (**C**) WB of H1299 transfected cells expressing constructs where the +1 AUG has been deleted (OvaΔATG and GAr-OvaΔATG) . (**D**) *In vitro* RNA coIP performed with recombinant NCL and mRNAs extracted from H1299 cells expressing *Ova*, OvaΔATG, GAr-Ova and GAr-OvaΔATG. The numbers indicate relative protein expression levels. The graph shows the relative fold change in NCL binding with the indicated mRNAs. Data presented are from three independent experiments.

In line with the scenario that nascent GAr peptide dislodges NAC from the ribosome and this results in the recruitment of NCL to the *GAr* RNA G4 structure, the interaction between NCL and the *GAr* RNA would require *GAr*-encoding mRNAs to be translated. We tested this by treating cells with cycloheximide (CHX) or harringtonine (Harr), two drugs that prevent mRNA translation. As expected, both drugs suppress the expression of GAr-Ova, Ova, and endogenous p21, while NCL and actin with longer half-life were less affected (Figure 5A). Importantly, *in vitro* RNA-coIP showed that the interaction between the *GAr-Ova* mRNA and NCL was reduced by ∼4-fold following CHX or Harr treatment. However, the NCL–*Ova* mRNA interaction was not affected by translation inhibitor treatments (Figure 5B). To further test if translation is needed to allow NCL to bind the *GAr*-encoding mRNA, we designed Ova and GAr-Ova constructs lacking AUG start codons (OvaΔATG and GAr-OvaΔATG) abrogating the expression of respective proteins (Figure 5C) and we observed that NCL had a reduced affinity for the *GAr-OvaΔATG* mRNA, as compared to the *GAr-Ova* mRNA (Figure 5D). The deletion of the AUGs had no significant effect on mRNA levels (Supplementary Figure S5A) or on their respective subcellular localisation (Supplementary Figure S5B and C). Thus, the *GAr*-encoding mRNAs need to be translated for binding NCL, in line with the notion that the interaction between NAC and the nascent GAr peptide is required for the recruitment of NCL to the G4 structure of the *GAr*-encoding mRNA on the EBNA1 polysome.

## DISCUSSION

Our data support a *cis*-acting protein-quality control pathway in which the disordered and aggregate-prone nascent gly-ala repeat (GAr) peptide of the EBNA1 dislodges NAC alpha (NACA) from the ribosome. NACA then interacts with the encoding *GAr* RNA sequence and helps recruit nucleolin (NCL) to the G-quadruplex (G4) structure of the *GAr* RNA, which leads to the suppression of mRNA translation initiation *in cis*. This illustrates how the EBV-encoded EBNA1 exploits a *cis*-acting cellular pathway for post-transcriptional gene regulation. The effect of the GAr on translation suppression is via initiation (26) and together with the fact that neither the RNA, nor the peptide, are degraded suggests that GAr-mediated translation control is a regulated process. For example, EBNA1 binds its own mRNA via a RGG-rich region and this could allow control of its own synthesis (36). EBNA1 is essential for viral survival but at the same time EBNA1 is immunogenic and this self-regulation of translation serves to minimize the production of antigenic peptides for the MHC class I pathway and at the same time guarantee a functional level of EBNA1 expression. The scenario in which the nascent GAr peptide serves as a trigger to suppress its own synthesis helps explain the observation that the GAr is more potent in suppressing synthesis when fused in the 5′ coding sequence rather than in the 3′ (Supplementary Figure S1E) (32). It is more likely that the presence of a disordered domain in the N-terminus, rather than the C-terminus, activates this pathway, in line with the fact that fusing a leader sequence to the N-terminus of an insoluble protein facilitates expression and solubility. There are few other examples of nascent peptides affecting protein expression but a more recent study on tubulin illustrates how the nascent peptide affects protein expression via the stability of the encoding mRNA, suggesting that the nascent peptide can activate different pathways.

We show that NACA binds the GAr peptide sequence as well as the encoding mRNA and, thus, providing the link between the GAr peptide and the encoding mRNA in controlling EBNA1 synthesis. *GAr* mRNA forms RNA G4 structures that interact with NCL and this is required for GAr-mediated translation initiation inhibition *in cis*. The observation that NACA binds the *GAr* G4 RNA structure and recruits NCL to the *GAr*-encoding RNA suggests that NACA makes the RNA structure NCL ‘friendly’. Previous studies have shown that the G4 RNA structures are highly dynamic *in vivo* with multi-functional properties and it is plausible that NACA affects the G4 structure to suit NCL binding (32). A similar ‘bind-unfold-lock’ mechanism was proposed for USP1 translational regulation by hnRNP H/F and DHX36 in glioblastoma or for the CNBP-targeted mRNAs (37,38).

An interesting aspect of NAC-mediated recruitment of NCL to the mRNA is where in the cell this event takes place. While NCL has a predominantly nucleolar localisation, NAC is present in the cytoplasm and the nuclear compartment. The PLA data show that the NACA – EBNA1 interaction takes place in the nucleus and it is an interesting possibility that this nascent peptide control pathway takes place during the ribosome-mediated RNA quality control that precedes nonsense-mediated decay.

The mechanism whereby the RNA and the encoded peptide act together via NAC to regulate mRNA translation *in cis* is specific in terms of both the RNA and the peptide sequence. Introducing a single serine in every eight residues of the GAr completely abolishes its translation inhibitory capacity (26) and introducing synonymous mutations also affects the efficacy of translation suppression (23). The GAr-mediated pathway of translation control is conserved in yeast (39). It is, however, plausible that this pathway reflects a specific example of a broader concept of regulating the synthesis of aggregate-prone proteins that includes the nascent peptide and the encoding RNA sequence but not necessarily via the same molecular mechanism.

It is not clear why both the RNA sequence and the encoding peptide should be required to suppress the synthesis of aggregate-prone proteins. One could argue that the sensing of a nascent disordered peptide should be sufficient to trigger suppression of synthesis in order to protect the cell from toxic aggregates. However, GC rich RNA sequences forming G4 structures, or disordered peptide domains in the N-terminus, are not uncommon and the fact that both peptide and RNA need to act together to trigger this pathway ensures that neither RNA sequence alone, nor peptide, will prevent synthesis. This could imply that aggregate-prone proteins that do not fulfil this double criterion would be more likely to be expressed at higher levels and accumulate aggregates. In the case of the aggregate-prone PolyQ of Huntingtin protein (HTT), the encoding RNA does not form G4 structures and it does not suppress translation when fused to the N-terminus of p53 (Supplementary Table S1) (26). It is also noteworthy that GC-rich sequences forming G4 structures encode peptides that can form aggregates, as is illustrated by patients suffering from Fragile X Syndrome. These patients have a (CGG) expansion that promotes alternative translation upstream of the classical *FMR1* ORF via repeat-associated non-AUG (RAN) (40,41). RAN is an alternative initiation mechanism mostly described for non-coding regions and introns and the encoded peptides cause toxic aggregates (42). Another example is from amyotrophic lateral sclerosis (ALS) which is associated with a GC-rich sequences from the (GGGGCC) repeats expansion in the *C9ORF72* gene (43). Both (CGG) and (GGGGCC) repeat expansions form G4 structures (43–45) and the encoded polypeptides are prone to form aggregates (46,47).

## DATA AVAILABILITY

All data are available upon request.

## Supplementary Material

gkac751_Supplemental_File

## References

[1] Candelise N. , Scaricamazza S., Salvatori I., Ferri A., Valle C., Manganelli V., Garofalo T., Sorice M., Misasi R. Protein aggregation landscape in neurodegenerative diseases: clinical relevance and future applications. Int. J. Mol. Sci. 2021; 22:6016. 34199513 10.3390/ijms22116016 PMC8199687

[2] Mogk A. , Bukau B., Kampinga H.H. Cellular handling of protein aggregates by disaggregation machines. Mol. Cell. 2018; 69:214–226. 29351843 10.1016/j.molcel.2018.01.004

[3] Goncalves C.C. , Sharon I., Schmeing T.M., Ramos C.H.I., Young J.C. The chaperone HSPB1 prepares protein aggregates for resolubilization by HSP70. Sci. Rep. 2021; 11:17139. 34429462 10.1038/s41598-021-96518-x PMC8384840

[4] Genest O. , Wickner S., Doyle S.M. Hsp90 and hsp70 chaperones: collaborators in protein remodeling. J. Biol. Chem. 2019; 294:2109–2120. 30401745 10.1074/jbc.REV118.002806 PMC6369297

[5] Mizuno M. , Ebine S., Shounai O., Nakajima S., Tomomatsu S., Ikeuchi K., Matsuo Y., Inada T. The nascent polypeptide in the 60S subunit determines the Rqc2-dependency of ribosomal quality control. Nucleic Acids Res. 2021; 49:2102–2113. 33511411 10.1093/nar/gkab005 PMC7913769

[6] Joazeiro C.A.P. Mechanisms and functions of ribosome-associated protein quality control. Nat. Rev. Mol. Cell Biol. 2019; 20:368–383. 30940912 10.1038/s41580-019-0118-2 PMC7138134

[7] Stein K.C. , Frydman J. The stop-and-go traffic regulating protein biogenesis: how translation kinetics controls proteostasis. J. Biol. Chem. 2019; 294:2076–2084. 30504455 10.1074/jbc.REV118.002814 PMC6369277

[8] Chandrasekaran V. , Juszkiewicz S., Choi J., Puglisi J.D., Brown A., Shao S., Ramakrishnan V., Hegde R.S. Mechanism of ribosome stalling during translation of a poly(A) tail. Nat. Struct. Mol. Biol. 2019; 26:1132–1140. 31768042 10.1038/s41594-019-0331-x PMC6900289

[9] Deuerling E. , Gamerdinger M., Kreft S.G. Chaperone interactions at the ribosome. Cold Spring Harb. Perspect. Biol. 2019; 11:a033977. 30833456 10.1101/cshperspect.a033977 PMC6824243

[10] Liu Y. , Hu Y., Li X., Niu L., Teng M. The crystal structure of the human nascent polypeptide-associated complex domain reveals a nucleic acid-binding region on the NACA subunit. Biochemistry. 2010; 49:2890–2896. 20214399 10.1021/bi902050p

[11] Gamerdinger M. , Kobayashi K., Wallisch A., Kreft S.G., Sailer C., Schlomer R., Sachs N., Jomaa A., Stengel F., Ban N. et al. Early scanning of nascent polypeptides inside the ribosomal tunnel by NAC. Mol. Cell. 2019; 75:996–1006. 31377116 10.1016/j.molcel.2019.06.030

[12] del Alamo M. , Hogan D.J., Pechmann S., Albanese V., Brown P.O., Frydman J. Defining the specificity of cotranslationally acting chaperones by systematic analysis of mRNAs associated with ribosome-nascent chain complexes. PLoS Biol. 2011; 9:e1001100. 21765803 10.1371/journal.pbio.1001100 PMC3134442

[13] Wang S. , Sakai H., Wiedmann M. NAC covers ribosome-associated nascent chains thereby forming a protective environment for regions of nascent chains just emerging from the peptidyl transferase center. J. Cell Biol. 1995; 130:519–528. 7622554 10.1083/jcb.130.3.519 PMC2120527

[14] Martin E.M. , Jackson M.P., Gamerdinger M., Gense K., Karamonos T.K., Humes J.R., Deuerling E., Ashcroft A.E., Radford S.E. Conformational flexibility within the nascent polypeptide-associated complex enables its interactions with structurally diverse client proteins. J. Biol. Chem. 2018; 293:8554–8568. 29650757 10.1074/jbc.RA117.001568 PMC5986199

[15] Shen K. , Gamerdinger M., Chan R., Gense K., Martin E.M., Sachs N., Knight P.D., Schlomer R., Calabrese A.N., Stewart K.L. et al. Dual role of ribosome-binding domain of NAC as a potent suppressor of protein aggregation and aging-related proteinopathies. Mol. Cell. 2019; 74:729–741. 30982745 10.1016/j.molcel.2019.03.012 PMC6527867

[16] Kirstein-Miles J. , Scior A., Deuerling E., Morimoto R.I. The nascent polypeptide-associated complex is a key regulator of proteostasis. EMBO J. 2013; 32:1451–1468. 23604074 10.1038/emboj.2013.87 PMC3655472

[17] Fabbri L. , Chakraborty A., Robert C., Vagner S. The plasticity of mRNA translation during cancer progression and therapy resistance. Nat. Rev. Cancer. 2021; 21:558–577. 34341537 10.1038/s41568-021-00380-y

[18] Holcik M. , Sonenberg N. Translational control in stress and apoptosis. Nat. Rev. Mol. Cell Biol. 2005; 6:318–327. 15803138 10.1038/nrm1618

[19] Sysoev V.O. , Fischer B., Frese C.K., Gupta I., Krijgsveld J., Hentze M.W., Castello A., Ephrussi A. Global changes of the RNA-bound proteome during the maternal-to-zygotic transition in drosophila. Nat. Commun. 2016; 7:12128. 27378189 10.1038/ncomms12128 PMC4935972

[20] Garcia-Moreno M. , Noerenberg M., Ni S., Jarvelin A.I., Gonzalez-Almela E., Lenz C.E., Bach-Pages M., Cox V., Avolio R., Davis T. et al. System-wide profiling of RNA-Binding proteins uncovers key regulators of virus infection. Mol. Cell. 2019; 74:196–211. 30799147 10.1016/j.molcel.2019.01.017 PMC6458987

[21] Beaudoin J.D. , Novoa E.M., Vejnar C.E., Yartseva V., Takacs C.M., Kellis M., Giraldez A.J. Analyses of mRNA structure dynamics identify embryonic gene regulatory programs. Nat. Struct. Mol. Biol. 2018; 25:677–686. 30061596 10.1038/s41594-018-0091-z PMC6690192

[22] Lista M.J. , Martins R.P., Billant O., Contesse M.A., Findakly S., Pochard P., Daskalogianni C., Beauvineau C., Guetta C., Jamin C. et al. Nucleolin directly mediates epstein-barr virus immune evasion through binding to G-quadruplexes of EBNA1 mRNA. Nat. Commun. 2017; 8:16043. 28685753 10.1038/ncomms16043 PMC5504353

[23] Yin Y. , Manoury B., Fahraeus R. Self-inhibition of synthesis and antigen presentation by epstein-barr virus-encoded EBNA1. Science. 2003; 301:1371–1374. 12958359 10.1126/science.1088902

[24] Apcher S. , Daskalogianni C., Manoury B., Fahraeus R. Epstein barr virus-encoded EBNA1 interference with MHC class i antigen presentation reveals a close correlation between mRNA translation initiation and antigen presentation. PLoS Pathog. 2010; 6:e1001151. 20976201 10.1371/journal.ppat.1001151 PMC2954899

[25] Murat P. , Zhong J., Lekieffre L., Cowieson N.P., Clancy J.L., Preiss T., Balasubramanian S., Khanna R., Tellam J. G-quadruplexes regulate epstein-barr virus-encoded nuclear antigen 1 mRNA translation. Nat. Chem. Biol. 2014; 10:358–364. 24633353 10.1038/nchembio.1479 PMC4188979

[26] Apcher S. , Komarova A., Daskalogianni C., Yin Y., Malbert-Colas L., Fahraeus R. mRNA translation regulation by the gly-ala repeat of epstein-barr virus nuclear antigen 1. J. Virol. 2009; 83:1289–1298. 19019958 10.1128/JVI.01369-08 PMC2620890

[27] Kikin O. , D’Antonio L., Bagga P.S. QGRS mapper: a web-based server for predicting G-quadruplexes in nucleotide sequences. Nucleic Acids Res. 2006; 34:W676–W682. 16845096 10.1093/nar/gkl253 PMC1538864

[28] Hon J. , Marusiak M., Martinek T., Kunka A., Zendulka J., Bednar D., Damborsky J. SoluProt: prediction of soluble protein expression in escherichia coli. Bioinformatics. 2021; 37:23–28. 10.1093/bioinformatics/btaa1102 PMC8034534 33416864

[29] Martins R.P. , Malbert-Colas L., Lista M.J., Daskalogianni C., Apcher S., Pla M., Findakly S., Blondel M., Fahraeus R. Nuclear processing of nascent transcripts determines synthesis of full-length proteins and antigenic peptides. Nucleic Acids Res. 2019; 47:3086–3100. 30624716 10.1093/nar/gky1296 PMC6451098

[30] Apcher S. , Daskalogianni C., Lejeune F., Manoury B., Imhoos G., Heslop L., Fahraeus R. Major source of antigenic peptides for the MHC class i pathway is produced during the pioneer round of mRNA translation. Proc. Natl. Acad. Sci. U.S.A. 2011; 108:11572–11577. 21709220 10.1073/pnas.1104104108 PMC3136330

[31] McQuin C. , Goodman A., Chernyshev V., Kamentsky L., Cimini B.A., Karhohs K.W., Doan M., Ding L., Rafelski S.M., Thirstrup D. et al. CellProfiler 3.0: Next-generation image processing for biology. PLoS Biol. 2018; 16:e2005970. 29969450 10.1371/journal.pbio.2005970 PMC6029841

[32] Zheng A.J. , Thermou A., Guixens Gallardo P., Malbert-Colas L., Daskalogianni C., Vaudiau N., Brohagen P., Granzhan A., Blondel M., Teulade-Fichou M.P. et al. The different activities of RNA G-quadruplex structures are controlled by flanking sequences. Life Sci. Alliance. 2022; 5:e202101232. 34785537 10.26508/lsa.202101232 PMC8605322

[33] Guo B. , Huang J., Wu W., Feng D., Wang X., Chen Y., Zhang H. The nascent polypeptide-associated complex is essential for autophagic flux. Autophagy. 2014; 10:1738–1748. 25126725 10.4161/auto.29638 PMC4198359

[34] Tovar Fernandez M.C. , Sroka E.M., Lavigne M., Thermou A., Daskalogianni C., Manoury B., Prado Martins R., Fahraeus R. Substrate-specific presentation of MHC class I-restricted antigens via autophagy pathway. Cell. Immunol. 2022; 374:104484. 35247713 10.1016/j.cellimm.2022.104484

[35] Meury T. , Akhouayri O., Jafarov T., Mandic V., St-Arnaud R. Nuclear alpha NAC influences bone matrix mineralization and osteoblast maturation in vivo. Mol. Cell. Biol. 2010; 30:43–53. 19884350 10.1128/MCB.00378-09 PMC2798287

[36] Lin Z. , Gasic I., Chandrasekaran V., Peters N., Shao S., Mitchison T.J., Hegde R.S. TTC5 mediates autoregulation of tubulin via mRNA degradation. Science. 2020; 367:100–104. 31727855 10.1126/science.aaz4352 PMC6942541

[37] Herviou P. , Le Bras M., Dumas L., Hieblot C., Gilhodes J., Cioci G., Hugnot J.P., Ameadan A., Guillonneau F., Dassi E. et al. hnRNP H/F drive RNA G-quadruplex-mediated translation linked to genomic instability and therapy resistance in glioblastoma. Nat. Commun. 2020; 11:2661. 32461552 10.1038/s41467-020-16168-x PMC7253433

[38] Benhalevy D. , Gupta S.K., Danan C.H., Ghosal S., Sun H.W., Kazemier H.G., Paeschke K., Hafner M., Juranek S.A. The human CCHC-type zinc finger nucleic acid-binding protein binds G-Rich elements in target mRNA coding sequences and promotes translation. Cell Rep. 2017; 18:2979–2990. 28329689 10.1016/j.celrep.2017.02.080 PMC5393907

[39] Lista M.J. , Voisset C., Contesse M.A., Friocourt G., Daskalogianni C., Bihel F., Fahraeus R., Blondel M. The long-lasting love affair between the budding yeast saccharomyces cerevisiae and the epstein-barr virus. Biotechnol. J. 2015; 10:1670–1681. 26311489 10.1002/biot.201500161

[40] Kearse M.G. , Wilusz J.E. Non-AUG translation: a new start for protein synthesis in eukaryotes. Genes Dev. 2017; 31:1717–1731. 28982758 10.1101/gad.305250.117 PMC5666671

[41] Todd P.K. , Oh S.Y., Krans A., He F., Sellier C., Frazer M., Renoux A.J., Chen K.C., Scaglione K.M., Basrur V. et al. CGG repeat-associated translation mediates neurodegeneration in fragile x tremor ataxia syndrome. Neuron. 2013; 78:440–455. 23602499 10.1016/j.neuron.2013.03.026 PMC3831531

[42] Cleary J.D. , Pattamatta A., Ranum L.P.W. Repeat-associated non-ATG (RAN) translation. J. Biol. Chem. 2018; 293:16127–16141. 30213863 10.1074/jbc.R118.003237 PMC6200949

[43] Fratta P. , Mizielinska S., Nicoll A.J., Zloh M., Fisher E.M., Parkinson G., Isaacs A.M. C9orf72 hexanucleotide repeat associated with amyotrophic lateral sclerosis and frontotemporal dementia forms RNA G-quadruplexes. Sci. Rep. 2012; 2:1016. 23264878 10.1038/srep01016 PMC3527825

[44] Ofer N. , Weisman-Shomer P., Shklover J., Fry M. The quadruplex r(CGG)n destabilizing cationic porphyrin TMPyP4 cooperates with hnRNPs to increase the translation efficiency of fragile x premutation mRNA. Nucleic Acids Res. 2009; 37:2712–2722. 19273535 10.1093/nar/gkp130 PMC2677883

[45] Kharel P. , Becker G., Tsvetkov V., Ivanov P. Properties and biological impact of RNA G-quadruplexes: from order to turmoil and back. Nucleic Acids Res. 2020; 48:12534–12555. 33264409 10.1093/nar/gkaa1126 PMC7736831

[46] Ash P.E. , Bieniek K.F., Gendron T.F., Caulfield T., Lin W.L., Dejesus-Hernandez M., van Blitterswijk M.M., Jansen-West K., Paul J.W.,3rd, Rademakers R. et al. Unconventional translation of C9ORF72 GGGGCC expansion generates insoluble polypeptides specific to c9FTD/ALS. Neuron. 2013; 77:639–646. 23415312 10.1016/j.neuron.2013.02.004 PMC3593233

[47] Mori K. , Arzberger T., Grasser F.A., Gijselinck I., May S., Rentzsch K., Weng S.M., Schludi M.H., van der Zee J., Cruts M. et al. Bidirectional transcripts of the expanded C9orf72 hexanucleotide repeat are translated into aggregating dipeptide repeat proteins. Acta Neuropathol. 2013; 126:881–893. 24132570 10.1007/s00401-013-1189-3

