# Supplementary material for: The nascent polypeptide-associated complex (NAC) controls translation initiation in cis by recruiting nucleolin to the encoding mRNA
Source: Nucleic Acids Res. 2022 Sep 15;50(17):10110–22. doi: 10.1093/nar/gkac751 (PMC9508830; doi:10.1093/nar/gkac751)
Supplement: gkac751_Supplemental_File [file gkac751_supplemental_file.pdf]

Supplementary Data

Peptide sequence	Length (nucleotides)	Predicted RNA G4s (no overlaps)	Length (amino acids)	Predicted solubility
GAr	708	27	236	0.382
polyQ	381	0	127	0.703
Half GAr	354	14	117	0.357

Table S1: Predicted solubility of the GAr and polyQ polypeptides. Predicted RNA G4 structures in the coding sequence of the mRNAs are identified using QGRS Mapper (71). Predicted solubilities for protein production in *E.coli* are calculated using the SoluProt v1.0 Webserver (72). For predicted solubility, a score below 0.5 indicates insoluble expression in *E.coli*.

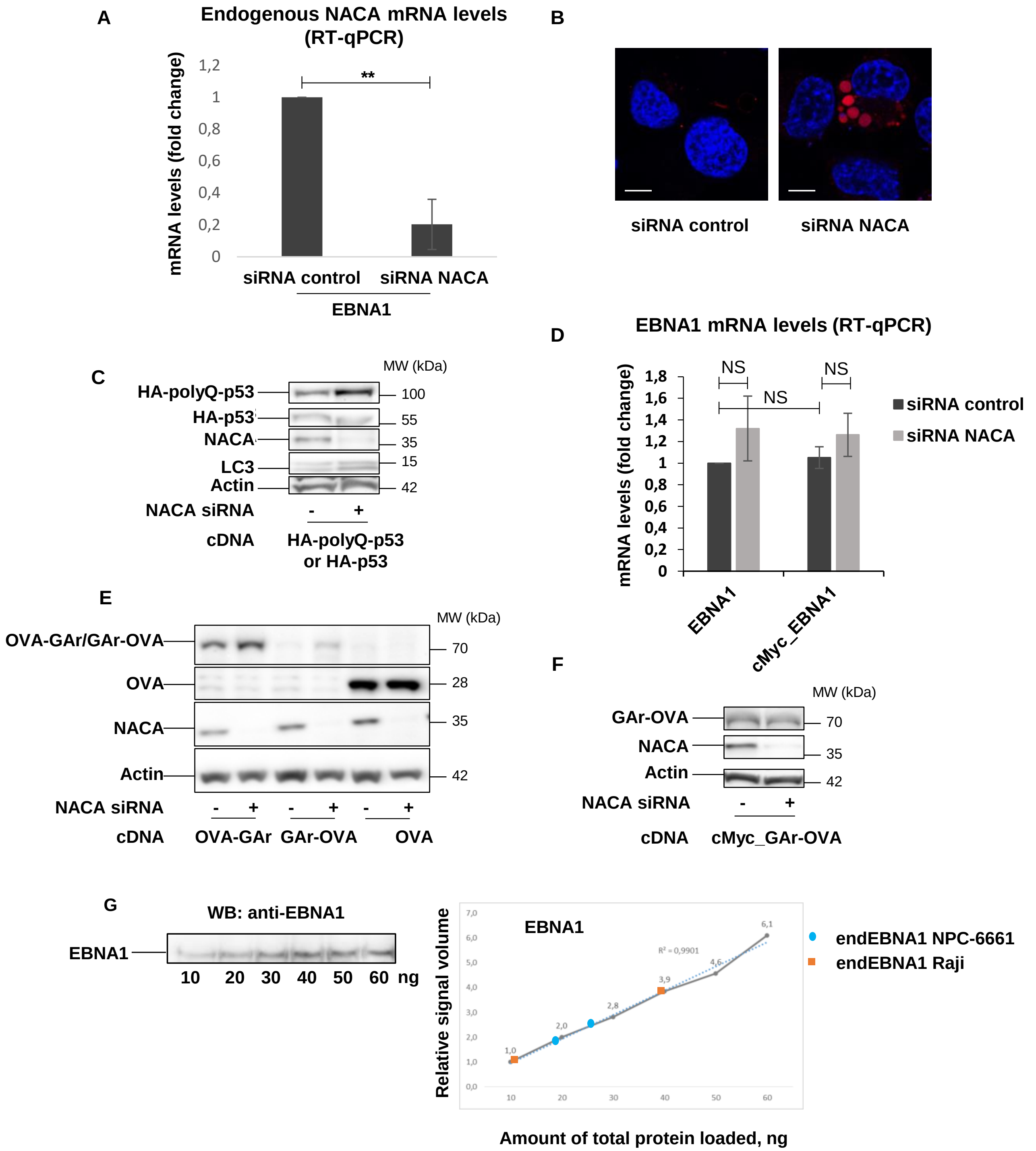

Supp. Fig. S1. **A.** Relative *NACA* mRNA levels determined by RT-qPCR from H1299 cells transfected with the EBNA1 construct, treated with siRNA control or siRNA against NACA. **B.** Microscopy images obtained with H1299 cells treated with siRNA control or siRNA against NACA, using the PROTEOSTAT® Aggresome Detection kit. Aggregates are coloured in red, nucleus in blue. Scale bar represents 10µm. **C.** WB from H1299 cells transfected with indicated constructs and treated with siRNA control or siRNA against NACA. **D.** Relative *EBNA1* mRNA levels determined by RT-qPCR from H1299 cells transfected with the EBNA1 or the cMyc_EBNA1 construct, treated with siRNA control or siRNA against NACA. **E.** WB from H1299 cells transfected with the indicated constructs, treated with siRNA control or siRNA against NACA. **F.** WB from H1299 cells transfected with the cMyc_GAr-Ova construct and treated with siRNA control or siRNA against NACA. **G.** Standard curves (right graph) of signal volume versus protein amount were generated using serial dilutions of EBNA1 containing lysate (WB, left). There is a strong linear correlation between signal volume and protein amount (R > 0,99) showing the reliable quantification of EBNA1 levels in NPC-6661 (in blue) and Raji cells (in red) (related to WBs in Fig. 1I). Data relate to figure 1.

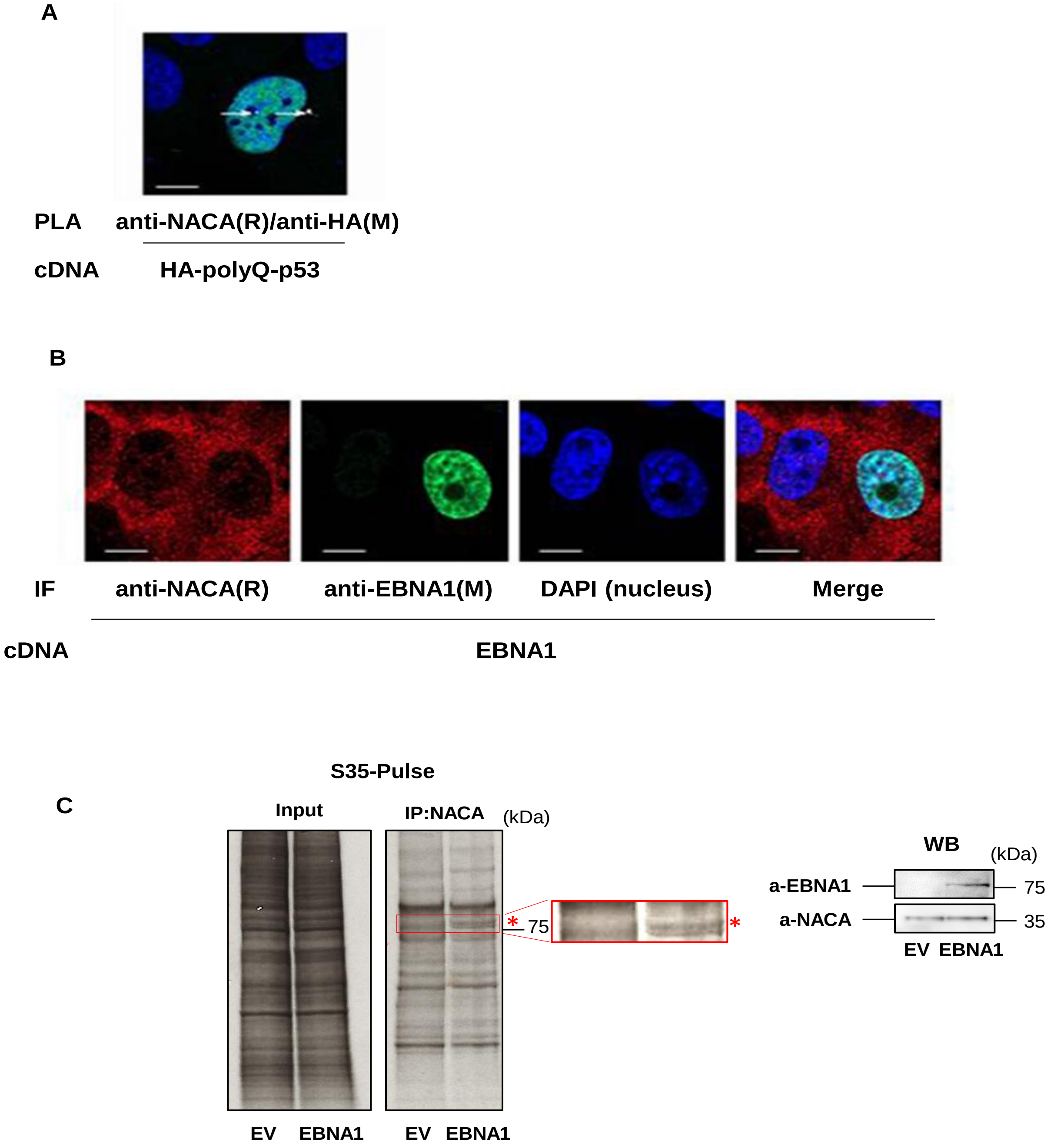

Supp. Fig. S2. **A**. PLA assessing NACA-HA interactions in H1299 cells transfected with HA-polyQ-p53 constructs, with co-immuno-staining of the reporter proteins. In blue: nucleus, in green: immuno-staining of the reporter proteins. White arrows and dots indicate PLA dots. Scale bar represents 10µm. **B**. Immuno-staining of H1299 cells expressing EBNA1 using antibodies against NACA (in red) or EBNA1 (in green). Nuclear staining (DAPI) in blue. (R):Rabbit; (M):Mouse antibodies. Scale bar represents 10µm. **C**. H1299 cells transfected with the indicated constructs were metabolically labelled with S35 methionine and immunoprecipitated using anti-NACA antibody. Co-immunoprecipitated newly synthesised proteins were separated with SDS-PAGE (IP:NACA). Input (S35 labeled proteins before the IP) is shown on the left. Right panel represents a WB of the lysates used for the metabolic labelling. Data relate to figure 2.

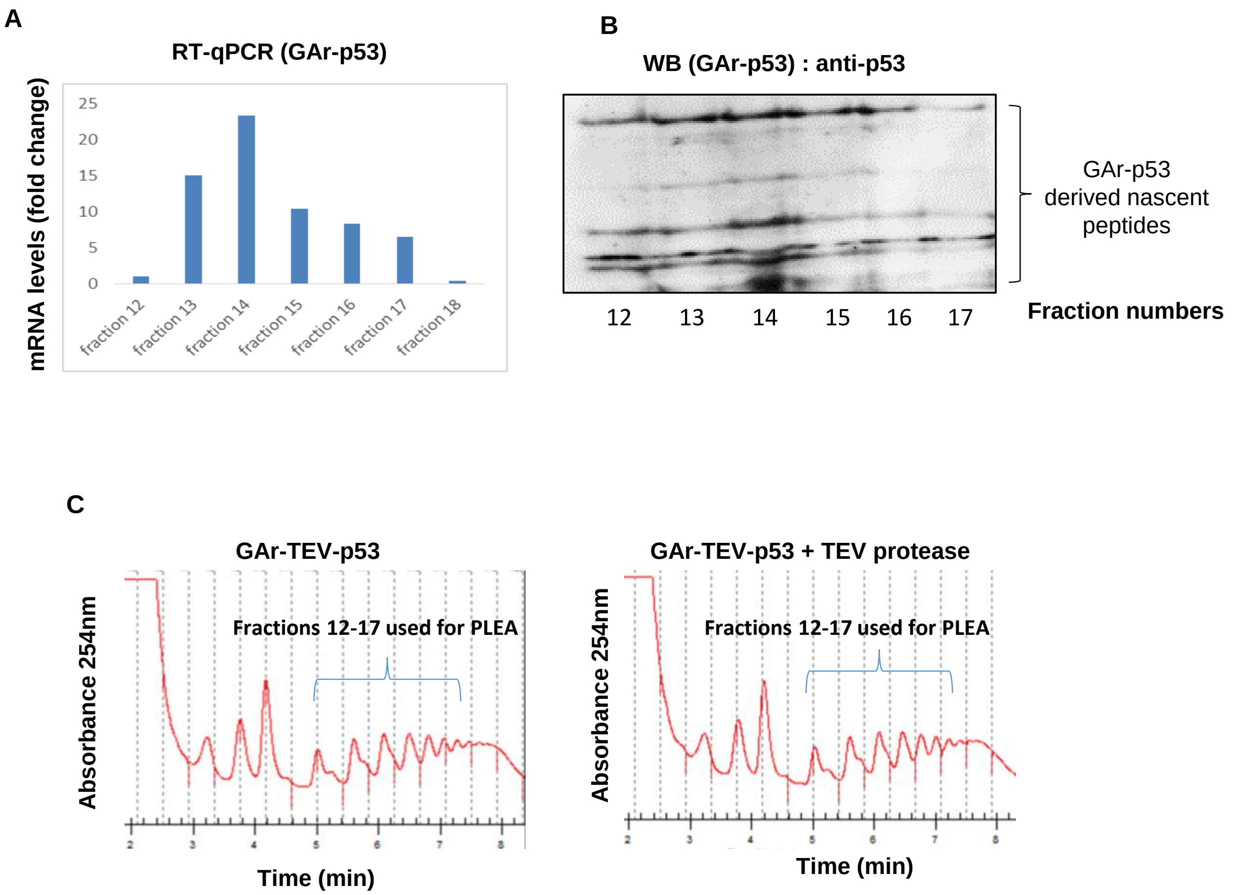

Supp. Fig. S3. A. RT-qPCR on the GAr-p53 mRNA extracted from polysomal fractions 12-17. **B.** WB from polysomal fractions 12-17 using anti-p53 antibody. **C.** Ribosomal profile of H1299 cells transfected with GAr-TEV-p53 construct and treated, or not, with TEV protease. Polysomal fractions 12-17 were used for PLEA experiments.Data relate to figure 3.

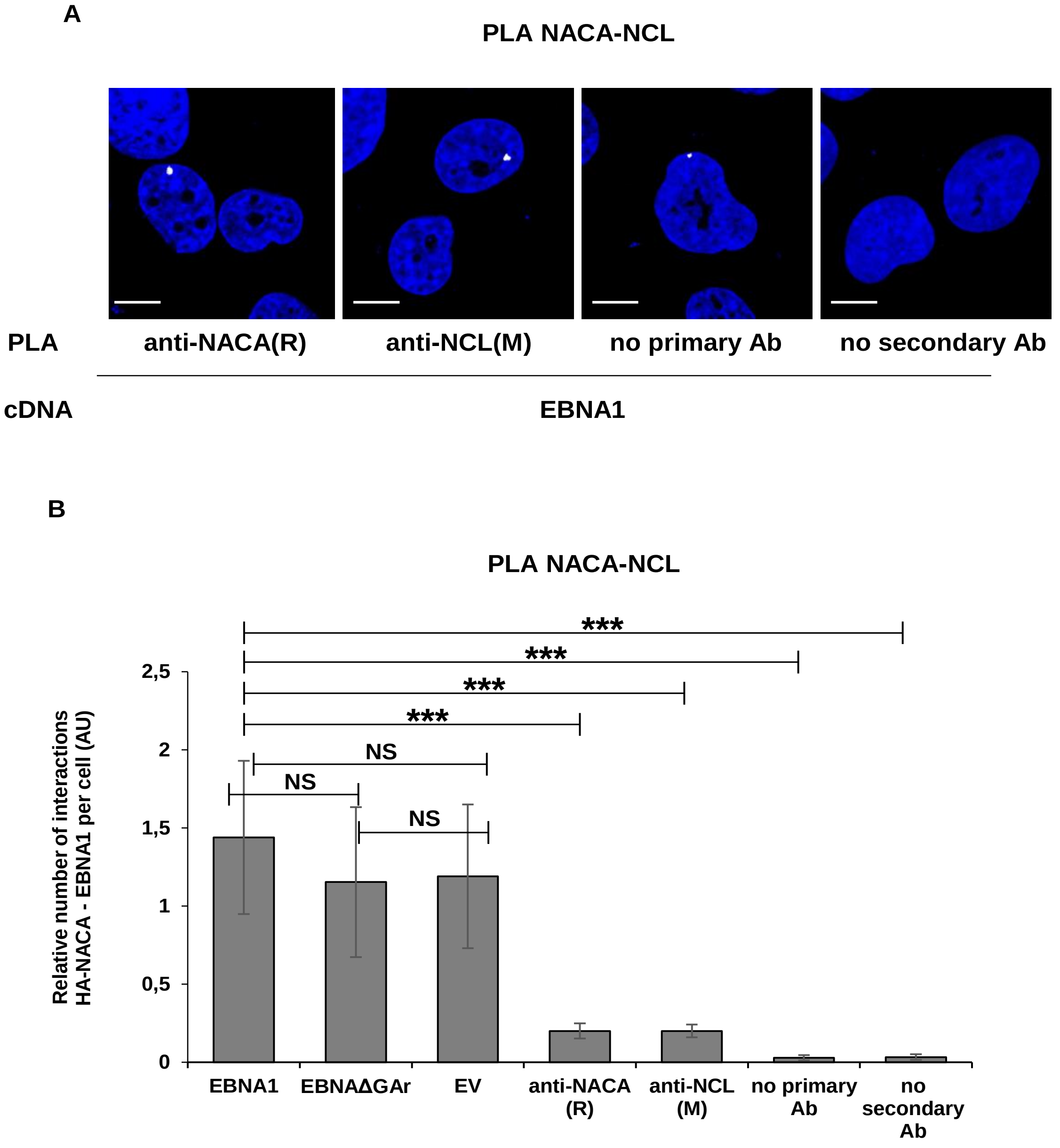

Supp. Fig. S4. A. Control conditions for the NACA - NCL PLA, where only the anti-NAC (1st image) or anti-NCL (2nd image) antibodies were used, or no primary antibody (3rd image) or no secondary antibody (4th image) was used. The figures show the occasional unspecific PLA signal. **B.** The graph represents relative number of interactions of the indicated reporter proteins with endogenous NACA detected by PLA and also the number of interactions in control conditions in non-transfected cells. (R):Rabbit; (M):Mouse antibodies. (R):Rabbit; (M):Mouse antibodies. Data relate to figure 4.

A

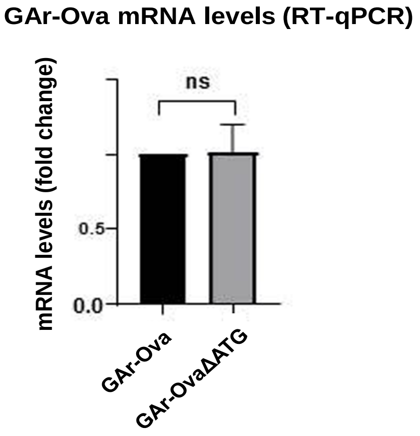

B

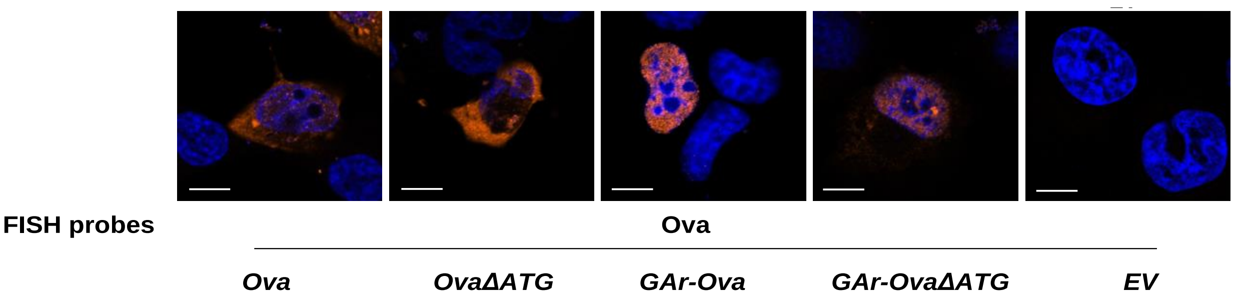

C

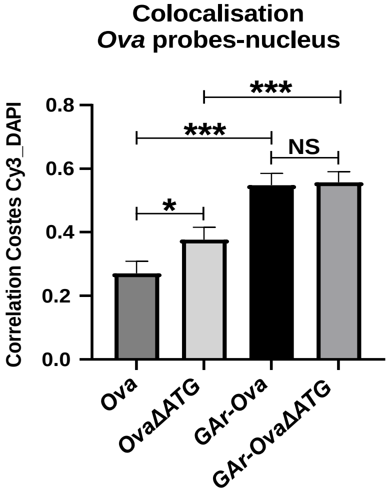

Supp. Fig. S5. A. Relative mRNA levels of *GAr-Ova* and *GAr-OvaΔATG* (lacks the 1st AUG) mRNAs as determined by RT-qPCR from H1299 cells. Graph shows data from three independent experiments. **B**. RNA FISH using Ova probes shows the subcellular localisation of *Ova, OvaΔATG, GAr-Ova, GAr-OvaΔATG* and empty vector (EV). **C.** Co- localisation of the *Ova, GAr-Ova*, *OvaΔATG* and *GAr-OvaΔnoATG* mRNAs with the nucleus using the mean Costes correlation factor for each condition (n=19 for GAr-Ova, 31 for GAr-OvaΔATG, 27 for Ova, 47 for OvaΔATG). A correlation factor of 1 indicates both signals perfectly co-localise. Data relate to figure 5.
